# BB0324 and BB0028 are constituents of the *Borrelia burgdorferi *β-barrel assembly machine (BAM) complex

**DOI:** 10.1186/1471-2180-12-60

**Published:** 2012-04-20

**Authors:** Tiffany R Lenhart, Melisha R Kenedy, Xiuli Yang, Utpal Pal, Darrin R Akins

**Affiliations:** 1 Department of Microbiology and Immunology, University of Oklahoma Health Sciences Center, Oklahoma City, OK 73104, USA; 2 Department of Veterinary Medicine, University of Maryland, College Park, MD 20742, USA; 3 Virginia-Maryland Regional College of Veterinary Medicine, College Park, MD 20742, USA; 4 Department of Botany and Microbiology, University of Oklahoma, Norman, OK 73019, USA

## Abstract

**Background:**

Similar to Gram-negative bacteria, the outer membrane (OM) of the pathogenic spirochete, *Borrelia burgdorferi*, contains integral OM-spanning proteins (OMPs), as well as membrane-anchored lipoproteins. Although the mechanism of OMP biogenesis is still not well-understood, recent studies have indicated that a heterooligomeric OM protein complex, known as BAM (β-barrel assembly machine) is required for proper assembly of OMPs into the bacterial OM. We previously identified and characterized the essential β-barrel OMP component of this complex in *B. burgdorferi*, which we determined to be a functional BamA ortholog.

**Results:**

In the current study, we report on the identification of two additional protein components of the *B. burgdorferi *BAM complex, which were identified as putative lipoproteins encoded by ORFs BB0324 and BB0028. Biochemical assays with a BamA-depleted *B. burgdorferi *strain indicate that BB0324 and BB0028 do not readily interact with the BAM complex without the presence of BamA, suggesting that the individual *B. burgdorferi *BAM components may associate only when forming a functional BAM complex. Cellular localization assays indicate that BB0324 and BB0028 are OM-associated subsurface lipoproteins, and *in silico *analyses indicate that BB0324 is a putative BamD ortholog.

**Conclusions:**

The combined data suggest that the BAM complex of *B. burgdorferi *contains unique protein constituents which differ from those found in other proteobacterial BAM complexes. The novel findings now allow for the *B. burgdorferi *BAM complex to be further studied as a model system to better our understanding of spirochetal OM biogenesis in general.

## Background

*Borrelia burgdorferi*, the spirochetal agent of Lyme disease, possesses a dual-membraned (diderm) architecture, which is composed of a peptidoglycan layer associated with the inner membrane (IM) and an outer membrane (OM) [1,2]. In Gram-negative bacteria, cytoplasmic precursor outer membrane proteins (OMPs) are synthesized with an amino-terminal signal peptide sequence, which typically targets a protein for Sec-mediated translocation. After the precursor OMP crosses the IM through the SecYEG translocase, the signal peptide is cleaved by signal peptidase I, and the mature protein is subsequently released into the periplasmic space [3-5]. Once in the periplasm, the unfolded OMP is bound by chaperones that help direct the OMP to the OM for proper folding and membrane insertion [6-8]. Until recently, these latter steps of periplasmic OMP trafficking and OM assembly have remained largely uncharacterized. In 2003, however, Tommassen and coworkers identified an essential β-barrel OMP whose function is dedicated to the proper OM-assembly of most known OMPs [9]. This protein, now known as BamA [10,11], is evolutionarily well-conserved since putative orthologs can be found in all known diderm bacteria, as well as in dual-membraned eukaryotic organelles, such as mitochondria and chloroplasts [7,12-15]. The functional importance of BamA was illustrated when researchers discovered that BamA was essential for the viability of both *N. meningitidis *and *E. coli*, and that its depletion resulted in dramatically decreased levels of properly-inserted OMPs in the OM of both organisms [9,16,17].

In *E. coli*, combined genetic and biochemical studies have now revealed that BamA exists in a multiprotein OM complex, termed the beta-barrel assembly machine (BAM) [10,11]. This complex is composed of the OM-imbedded BamA protein and four OM-anchored accessory lipoproteins, termed BamB, BamC, BamD, and BamE (previously known as YfgL, NlpB, YfiO, and SmpA respectively) [10,18-20]. More recent studies have revealed that all of the BAM components are important at some level for OMP assembly and/or for the stability of the BAM complex. The BamB lipoprotein interacts directly with BamA within the complex, and this association is independent of the other BAM lipoproteins [19,21]. BamB is thought to be an important scaffolding protein for the BAM complex, and although BamB deletion mutants are viable, they have reduced levels of various OMPs [20,22-26]. *bamC*- and *bamE*-null strains have relatively mild OMP assembly defects; however, they both show moderate OM permeability defects, and biochemical studies show that their presence in the complex is important for the BamA-BamD interaction [18,19,21,25]. The BamD protein, however, is essential for cell viability, and depletion of BamD causes a phenotype similar to that observed in BamA mutants [21,25]. Additionally, BamD is the most evolutionarily conserved lipoprotein in the BAM complex. Like BamA, BamD orthologs are predicted to be present in all diderm bacteria [6,15,21], and they are proposed to contain conserved tetratricopeptide repeat (TPR) domains which have been shown to function in protein-protein interactions [27-29].

BAM complexes have now been characterized from other Gram-negative bacteria, such as *N. meningitidis *and *Caulobacter crescentus *[30,31]. In *N. meningitidis*, co-immunoprecipitation studies showed that, along with BamA and BamD, the BAM complex contains BamC and BamE, as well as an additional protein component, RmpM [30]. A BamB homolog, however, was not identified in *N. meningitidis*. The BAM complex in *C. crescentus *was recently reported to contain all of the known BAM lipoproteins except BamC, but includes an additional lipoprotein termed Pal, which contains an OmpA-type peptidoglycan binding domain that is similar to RmpM [31]. These studies suggest that bacterial BAM complexes likely contain not only conserved orthologs and proteins with conserved structural motifs, such as BamD, but also non-conserved proteins which may provide specific requirements for OMP assembly in a particular species of bacteria.

In *B. burgdorferi*, the only member of the BAM complex identified to date is BB0795, which we previously determined to be a structural and functional *B. burgdorferi *BamA ortholog [32]. In the present study, we examined whether *B. burgdorferi *BamA, like other known BamA proteins, exists as a member of a multiprotein OM complex. We report that native *B. burgdorferi *BamA forms high molecular-weight OM complexes and that BamA co-immunoprecipitates specifically with two putative *B. burgdorferi *lipoproteins, BB0324 and BB0028. We also demonstrate that depletion of BamA, using an IPTG-regulated *B. burgdorferi *mutant, results in loss of BB0324-BB0028 interactions, suggesting that the lipoproteins do not associate without the presence of BamA. Additionally, we determined that both BB0324 and BB0028 are OM-anchored, and are localized to the inner leaflet of the OM. While sequence analysis strongly suggests that BB0324 is a BamD ortholog containing TPR domains similar to those predicted for the *N. meningitidis *and *E. coli *BamD lipoproteins [15], BB0028 did not have significant sequence homology to any other known BAM components. The combined results suggest that *B. burgdorferi *contains fewer proteins in its BAM complex, which is likely reflective of its distinct evolutionary phylogeny and unique OM ultrastructure.

## Methods

### Bacterial strains and growth conditions

*Borrelia burgdorferi *strain B31-MI, strain B31-A3 [33], strain B31-A3-LK [34], and strain *flacp*-795-LK [32] were cultivated at 34°C in Barbour-Stoenner-Kelly (BSK-II) liquid medium [35] containing 6% heat-inactivated rabbit serum (complete BSK-II). The B31-A3 strain was supplemented with kanamycin (200 μg/mL), and the B31-A3-LK strain was supplemented with kanamycin and gentamicin (40 μg/mL). Strain *flacp*-795-LK was supplemented with 100 μg/mL streptomycin (selection for the *flacp *regulatable promoter), in addition to kanamycin and gentamycin. Strain *flacp*-795-LK was also cultivated in 0.05 mM or 1.0 mM isopropyl-β-D-thiogalactopyranoside (IPTG), as indicated.

### Isolation of *B. burgdorferi *outer membrane vesicles and protoplasmic cylinders

For Blue Native PAGE (BN-PAGE) and cellular localization assays, *B. burgdorferi *strain B31-A3 outer membranes (OMs) and protoplasmic cylinders (PCs) were isolated by discontinuous sucrose density gradient centrifugation followed by a continuous sucrose density gradient centrifugation protocol as previously described [32,36].

### Blue native PAGE (BN-PAGE) analysis

*B. burgdorferi *strain B31-A3 OM complexes were analyzed by BN-PAGE under native conditions as described [37,38]. Briefly, the isolated OM preparations were resuspended in 0.75 M aminocaproic acid, 50 mM Bis-Tris (pH 7.0) and β-dodecyl maltoside (DM) (DM/protein = 40 w/w). The protein solution was incubated for 30 min on ice and centrifuged at 14,000 × *g *for 30 min, and the resulting supernatant was separated using a 5-14% gradient polyacrylamide gel at 4°C. The protein migration pattern in the BN gel was analyzed visually, or electrophoretically transferred to nitrocellulose for anti-BamA immunoblot analysis, as described below.

### SDS-PAGE and immunoblot analyses

For denaturing PAGE and immunoblots, protein samples were prepared and separated by SDS-PAGE, followed by electrophoretic transfer to nitrocellulose membranes, as described previously [32]. For FlaB immunoblots, membranes were probed with a 1:2,000 dilution of rabbit anti-FlaB antisera [39], followed by incubation with a 1:2,000 dilution of horseradish peroxidase (HRP)-conjugated goat anti-rabbit secondary antibodies (Invitrogen, Carlsbad, CA). Subsequent chromogenic development was performed using 4-chloronapthol and hydrogen peroxide. For all other immunoblots, enhanced chemiluminescence (ECL) was used, as described by Kenedy *et al. *[40]. After primary antibody incubation [BamA, BB0405, and OppAIV (1:2,000); BB0324, BB0028, and Lp6.6 (1:5,000); OspA (1:100,000)], membranes were incubated in a 1:10,000 dilution of goat anti-rat (for BamA, BB0324, BB0405, OspA, and OppAIV blots), goat anti-rabbit (for BB0028 blots), or goat anti-mouse (for Lp6.6 blots) secondary antibodies. Washed membranes were subsequently developed using SuperSignal West Pico ECL reagent according to manufacturer's instructions (Thermo Fischer Scientific, Inc., Rockford, IL).

### Sequence analyses and alignments

The *N. meningitidis *BamD (Nm-BamD) protein sequence was used to search the *B. burgdorferi *B31 peptide database using the J. Craig Venter Comprehensive Microbial Resource Blast server (http://blast.jcvi.org/cmr-blast/). BB0324 and BB0028 hydrophilicity analyses were performed using MacVector version 10.0 sequence analysis software (MacVector, Inc., Cary, NC) according to the method of Kyte and Doolittle [41], and prediction of putative signal peptides and the canonical lipoprotein signal peptidase II cleavage sites was performed using the SignalP 3.0 server [42,43] and the LipoP 1.0 server [44], respectively. BB0324 tetratricopeptide repeat (TPR) domains were predicted using TPRpred (http://toolkit.tuebingen.mpg.de/tprpred) and by comparison with the original published TPR consensus sequence [27]. The predicted TPR-containing regions from Nm-BamD, *E*. coli BamD, and BB0324 (residues 35-106, residues 32-102, and residues 28-100, respectively) were aligned using the MacVector version 10.0 multiple sequence alignment program (MacVector, Inc.), followed by manual editing using the Jalview 2 multiple alignment editor [45].

### Generation and purification of recombinant proteins

To generate BB0324, BB0796, and BB0028 recombinant proteins, DNA sequences corresponding to each full-length mature protein lacking the putative signal peptide were PCR-amplified from B31 genomic DNA. Primers used for amplification of the *bb0324 *DNA region are as follows (restriction sites are indicated in bold): 5'-GCG**GGATCC**TTAACAAAAGAAACTCCTTATGG-3' (*BamH*I site plus nucleotides 64 to 68), and 5'-TTTTTTATTATTTTCTATTTTATTTAATA-3' (complementary to nucleotides 357 to 329). Primers used for amplification of the *bb0796 *DNA region are as follows: 5'-GCG**GGATCC**GCTAATCTTGATCAAATAAAAAATC-3' (*BamH*I site plus nucleotides 151 to 175) and 5'-GCGGAATCCTTAAGGGTTTTTATTGTCCTTTTC-3' (complementary to nucleotides 558 to 535 plus the *EcoR*I site). Primers used for amplification of the *bb0028 *DNA region are as follows: 5'-AA**GAATTC**TCAAGCGAATCCATATTTTCAC-3' (*EcoR*I site plus nucleotides 76 to 98), and 5'-AA**CTCGAG**TTATTCTTTAGTTAATTTTCTGTTTTCCA-3' (complementary to nucleotides 1050 to 1021 plus the *Xho*I site). The *bb0324, bb0796*, and *bb0028 *amplicons were ligated into the Topo-TA pBAD/Thio vector (Invitrogen), the pGEX-4 T-3 vector (GE Healthcare, Piscataway, NJ), and the pGEX-6P1 vector (GE Healthcare), respectively. The resulting constructs were transformed into electrocompetent *E. coli *DH5α cells, and prior to protein purification, selected transformants were verified to contain the correct insert sequence by restriction digest and by nucleotide sequence analysis.

For protein purification, recombinant BB0324 was purified as a thioredoxin fusion using a solubilization protocol described previously [32]. Recombinant BB0796 and BB0028 were purified as glutathione-*S*-transferase (GST) fusion proteins and cleaved free of the GST moiety using procedures described previously [46-48].

### Antibodies

Antibodies to the BB0324 and BB0796 recombinant proteins were generated in rats as previously described [32,39]. Rabbit anti-BB0028 antibodies were described elsewhere [49]. Rat anti-BamA (BB0795) antibodies were generated previously [32], and mouse anti-Lp6.6 antibodies were also generated as described previously [37]. Mouse anti-OppAIV antibodies were generously provided by Drs. Justin Radolf and Melissa Caimano, University of Connecticut Health Center, Farmington, CT. Rabbit anti-FlaB, rat anti-Thio, rat anti-OspA, and rat anti-405 antibodies were generated as previously described [39,50]. All animal procedures were approved by the Oklahoma University Health Sciences Center Institutional Animal Care and Use Committee (protocol # 07-128).

### Cell lysate preparation and co-immunoprecipitation (co-IP)

For each co-IP sample, cell lysates were prepared by using mid-log phase cultures (2 × 10^10 ^organisms) of *B. burgdorferi *strain B31-MI, B31-A3-LK, or *flacp*-795-LK (grown in either 0.05 mM or 1.0 mM IPTG). Cells were centrifuged at 5,000 × *g *for 20 min and subsequently washed four times in PBS (pH 7.4). Prior to cell lysis for co-IP, washed cells (4 × 10^7 ^organisms) from each culture condition were subjected to anti-BamA immunoblot analysis to verify the regulatable BamA phenotype. For co-IP experiments, cell pellets were solubilized and lysed by resuspension in 1× BugBuster Reagent (EMD Biosciences, Inc., Darmstadt, Germany; 2.5 mL per gram of wet cell weight). The solubilized cell solution was supplemented with 2 μL Lysonase Bioprocessing Reagent (EMD Biosciences, Inc.) and 20 μL of protease inhibitor cocktail (Sigma Chemical Company, St. Louis, MO) per co-IP sample, and the mixture was subsequently rocked at room temperature (RT) for 20 min. Finally, the cell debris was pelleted at 15,000 × *g *for 15 min at 4°C, and the supernatant (containing the cell lysate) was used for the co-IP experiments.

Co-IPs were performed using the Sigma Protein G Immunoprecipitation Kit according to manufacturer's instructions, with the following modifications: 1) the 1× and 0.1× IP Buffers were supplemented with 0.2% Triton X-100, and 2) prior to immunoprecipitation, the lysates were pre-cleared overnight to reduce background binding. After immunoprecipitation, bound proteins were eluted in 50 μL final sample buffer [62 mM Tris-HCl (pH 6.8), 10% v/v glycerol, 100 mM DTT, 2% SDS, 0.001% bromophenol blue], subjected to SDS-PAGE, and analyzed by silver stain according to the procedure of Morrissey [51], or by immunoblot, as described above.

For protein identification, excised SDS-PAGE gel bands were submitted to the Molecular Biology-Proteomics Facility (University of Oklahoma HSC, Oklahoma City, OK) for tryptic digestion and HPLC-MS/MS analysis, followed by MASCOT database search for protein identification.

### Triton X-114 (TX-114) phase partitioning

To determine whether BB0324 and BB0028 have the amphipathic properties of typical lipid-modified proteins, *B. burgdorferi *strain B31-MI cells (2 × 10^8 ^organisms) were harvested and phase-partitioned as described previously [39,52].

### Proteinase K (PK) surface accessibility

To determine whether BB0324 and BB0028 contain surface-exposed regions, PK experiments were performed as previously described [39]. Briefly, spirochetes (2 × 10^8 ^organisms) were harvested at 4,000 × *g*, washed four times in 1× PBS (pH 7.4), and the washed cells were either mock-treated or PK-treated (400 μg/μl); Sigma Chemical Co.) for one hour at RT. After addition of PMSF (0.4 mM final concentration), samples were prepared for SDS-PAGE and immunoblot analysis, as described above. To verify that BB0324 and BB0028 were not resistant to PK activity, cell membranes were disrupted as previously described [53]. Cells (2 × 10^8 ^or 1 × 10^9^) were pelleted at 10,000 × *g*, washed, and incubated for 10 m in 200 μl PK lysis buffer containing 50 mM Tris, 0.5% Triton X-100, 0.1%, β-mercaptoethanol, and 50 μg of lysozyme. Samples were then incubated in the absence or presence of PK. The reaction was stopped with PMSF and prepared for immunoblot as indicated above.

## Results

### *B. burgdorferi *BamA forms multi-protein complexes in the OM

**Figure 1:**
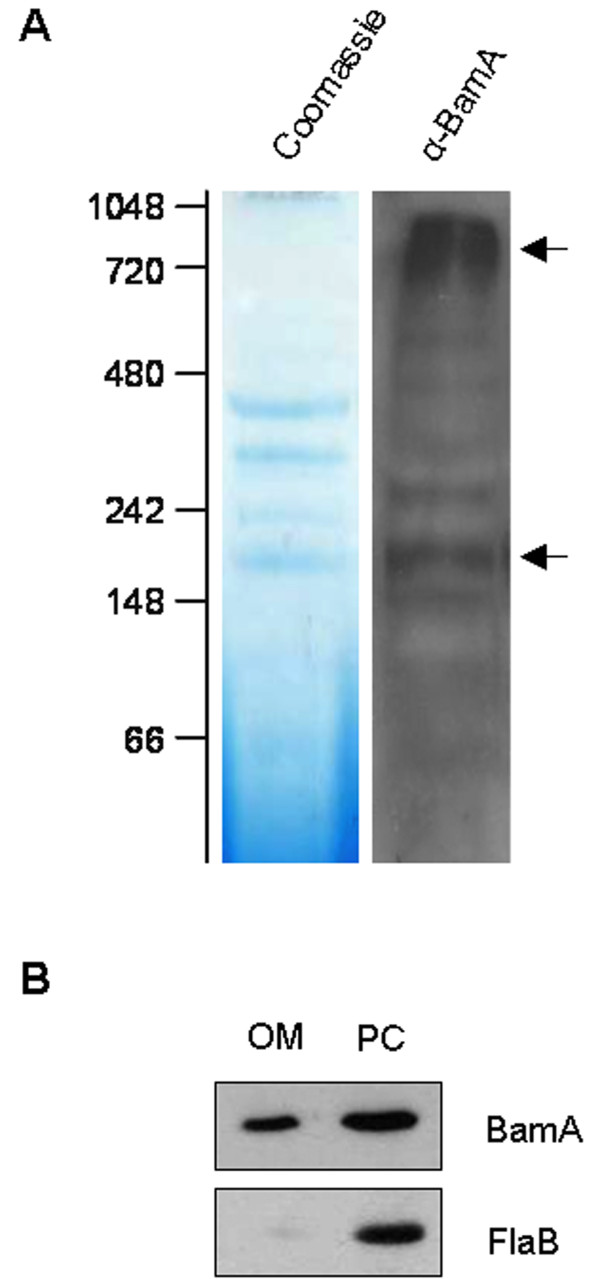
***B. burgdorferi *BamA is present in OM protein complexes**. A. The presence of BamA in OM complexes was revealed by blue native (BN)-PAGE analysis. OM proteins (20 μg) were separated by one-dimensional BN-PAGE (left panel). Subsequently, a strip of BN gel was excised and electrophoretically transferred, and immunoblot analysis was performed with anti-BamA antisera (right panel). Molecular weight standards, in kDa, are indicated at left. Arrows indicate two prominent bands resolving at ~200 kDa and 1000 kDa. **B**. Purity of a representative OM preparation used for BN analysis. *B. burgdorferi *protoplasmic cylinders (PCs) and OMs were isolated by sucrose density gradient centrifugation, as described in Methods. Cell equivalents of OM and PC fractions were separated by SDS-PAGE, electrophoretically transferred onto nitrocellulose membrane, and subsequently immunoblotted with antibodies against BamA and the periplasmic FlaB protein. As expected, BamA is present in the OM, while FlaB is enriched only in the PC fraction.

Previously, we performed a structural and functional characterization of the OM-localized *B. burgdorferi *BamA protein [32]. Since other BamA orthologs are known to exist in a hetero-oligomeric protein complex [10,18,20,30,31], we wanted to determine if native *B. burgdorferi *BamA could be detected in high molecular weight OM complexes. To perform this assay, we isolated OM vesicles from *B. burgdorferi *strain B31-A3 and subjected the OM sample to one-dimensional blue native (BN)-PAGE, followed by anti-BamA immunoblot analysis. Results from the immunoblot showed multiple protein bands between the 148 and 1,048 kDa MW markers (Figure 1A), with two prominent bands that resolved at approximately 200 kDa and 1,000 kDa (Figure 1A, arrows). In addition, samples from the OM fraction and from the protoplasmic cylinder (PC) fraction were separated by denaturing SDS-PAGE and immunoblotted against the periplasmic FlaB protein to verify OM purity (Figure 1B). These results demonstrate that native *B. burgdorferi *BamA is present in multiple high molecular weight OM complexes, which may indicate that BamA associates with other OM-localized proteins or protein complexes.

### *In silico *analysis of *B. burgdorferi *BAM orthologs

To identify possible components of the *B. burgdorferi *BAM complex, our initial approach was to search the *B. burgdorferi *protein database for putative orthologs of the *E. coli *BAM lipoproteins, BamB, BamC, BamD, and BamE [18]. Although protein Blast (BlastP) searches using each of the BAM proteins provided no significant sequence matches, BlastP searches using each of the *N. meningitidis *BAM lipoproteins as a search query yielded one *B. burgdorferi *protein. This protein, encoded by open reading frame (ORF) *bb0324*, has significant similarity (P value = 7.2 × 10^-5^) to the *N. meningitidis *BamD lipoprotein. BB0324 is a 119-residue polypeptide of unknown function that is predicted to contain an N-terminal signal peptide with a signal peptidase II lipoprotein modification and processing site as determined by a combination of hydrophilicity, SignalP 3.0, and LipoP 1.0 computer analyses as described in Methods. The identification of a canonical lipoprotein processing and modification site strongly suggested that BB0324 is the *B. burgdorferi *lipoprotein BamD ortholog.

**Figure 2:**
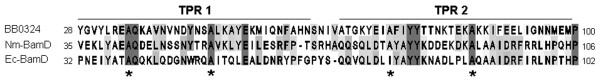
**Alignment of BB0324 and the BamD TPR domains**. Amino acid alignments of the N-terminal TPR (tetratricopeptide repeat) domains of *B. burgdorferi *BB0324, *N. meningitidis *BamD, and *E. coli *BamD. Each protein is predicted to contain two 34-residue TPR domains (indicated above alignments), with the amino acid positions of the TPR regions labeled at both the N- and C-termini. Amino acids are shaded based on sequence similarity, with the darkest shade indicating residues that are conserved among all three aligned sequences. The conserved TPR consensus sequence contains an Ala at positions 8 and 20, as indicated by asterisks. Note that the *B. burgdorferi *and *N. meningitidis *BamD proteins have these highly conserved residues in their TPR 1 and 2 motifs.

Comparative sequence analyses indicate that BB0324 aligns with the N-terminus of *N. meningitidis *BamD, such that almost the entire BB0324 amino acid sequence aligns with the first 100 residues of the 267-residue *N. meningitidis *BamD protein (Figure 2). Importantly, this region of *N. meningitidis *BamD is predicted to contain two conserved TPR sequences, which are also predicted to exist in BB0324 (indicated in Figure 2). The TPR sequence is a degenerate 34-residue consensus sequence that forms a helix-turn-helix secondary structure element [27-29], and such motifs are known to be involved in protein-protein interactions [27-29]. Only a few positions within the consensus TPR sequence are highly conserved (*e.g*., typically Gly or Ala at the eighth position and Ala at position 20, indicated by asterisks in Figure 2), and therefore individual TPRs can vary substantially at the primary sequence level. *E*. coli BamD is also predicted to contain N-terminal TPR sequences that can be aligned with those of BB0324 and *N. meningitidis *BamD (Figure 2). The combined results from the protein blast searches and the sequence alignment analyses further support the contention that BB0324 is a *B. burgdorferi *BamD ortholog.

### *B. burgdorferi *BamA forms a complex with BB0324 and BB0028

**Figure 3:**
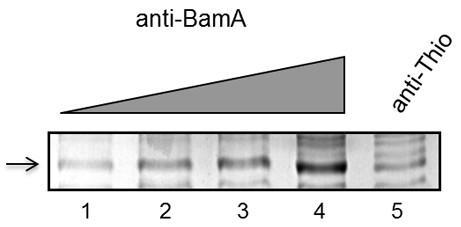
**SDS-PAGE analysis of anti-BamA co-IP elutions**. Cultures of *B. burgdorferi *strain B31 MI (2 × 10^10 ^organisms per sample) were washed and solubilized, and the protein-containing cell lysate was used for co-immunoprecipitation (co-IP) experiments using anti-Thio and anti-BamA polyclonal antibodies. Lanes 1-4 of the Coomassie-stained SDS-PAGE gel shows the 40 kDa region from elutions of anti-BamA co-IP experiments using increasing amounts (5 μL, 10 μL, 20 μL, or 40 μL) of antibody (titration indicated by grey triangle). An unknown protein that was enriched with increasing amount of anti-BamA antibody is indicated at left (arrow). A sample from the anti-Thio elutions (from which 40 μL antibody was used for co-IP) is shown in lane 5.

To identify additional BAM accessory proteins, we next performed anti-BamA co-immunoprecipation (co-IP) experiments. Since our BamA antisera was generated against recombinant BamA proteins with a 5' thioredoxin fusion (see Methods), we utilized anti-thioredoxin (anti-Thio) antisera as our negative control antibody for the co-IP assays. When we analyzed the immunoprecipitated proteins by coomassie stain, we detected an ~40 kDa band that was prominent in the BamA-precipitated samples as compared with the anti-Thio control (Figure 3). To determine the identity of the protein(s) contained within the 40 kDa band identified, this region (from both the BamA and the control Thio elutions, Figure 3 lanes 4 and 5, respectively) were subsequently excised, trypsin-digested, and subjected to LC-MS/MS analysis. After MASCOT database search, the unknown protein from the BamA co-IP was identified as a 349-residue polypeptide encoded by the *B. burgdorferi *ORF *bb0028*. This protein was not identified in the band extracted from the Thio co-IP elution, suggesting that it co-immunoprecipitated specifically with BamA. Similar to BB0324, computer analyses of the BB0028 protein indicated that it contains a signal peptide with a consensus signal peptidase II lipoprotein modification and processing site, suggesting that BB0028 is also a *B. burgdorferi *lipoprotein. Interestingly, BlastP analyses failed to identify any BB0028 conserved domains or any significant protein matches outside of the *Borrelia *genus.

**Figure 4:**
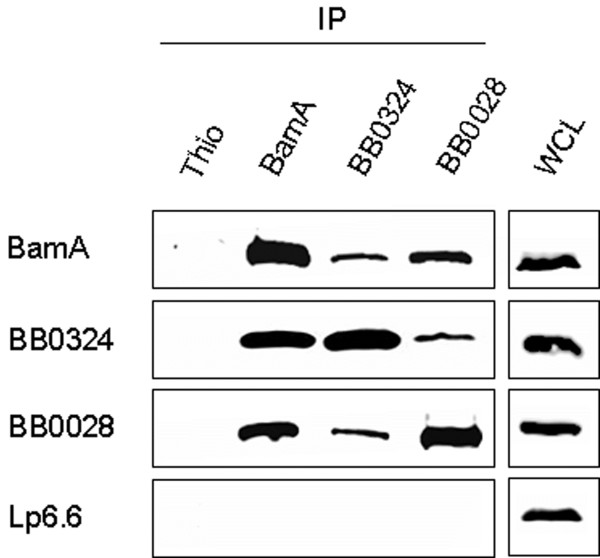
***B. burgdorferi *BamA, BB0324, and BB0028 co-immunoprecipitate (co-IP)**. Cultures of *B. burgdorferi *strain B31-MI (2 × 10^10 ^organisms per sample) were washed and solubilized, and the protein-containing cell lysate was used for co-IP experiments using anti-Thio, anti-BamA, anti-BB0324, and anti-BB0028 polyclonal antibodies (indicated above panels). Equal amounts of each co-IP elution were subjected to SDS-PAGE and immunoblot analysis using antisera generated against BamA, BB0324, and BB0028 (indicated at left of each panel). To illustrate specificity of the BamA-BB0324-BB0028 interaction, elutions were also immunoblotted with antibodies against an unrelated subsurface OM lipoprotein, Lp6.6 (bottom panel). Anti-Thio antibodies were used in the co-IP experiments as a negative control (left lane of each panel). Additionally, whole-cell lysates (WCL) were included as positive controls for the immunoblot procedure (right panels).

To determine if BB0324 (the putative BamD ortholog) and BB0028 are BAM accessory proteins that specifically associate with BamA, we performed anti-BamA, anti-BB0324, and anti-BB0028 immunoprecipitation experiments (Figure 4; antibodies used for immunoprecipitation assays are listed above panels). The immunoprecipitation assays were then subjected to immunoblot analysis with specific antibodies to BamA, BB0324, and BB0028 (indicated at left of panels). As shown in Figure 4, *B. burgdorferi *BamA co-immunoprecipitated BB0324 and BB0028. Additionally, BB0324 antibodies co-immunoprecipitated BamA and BB0028 while BB0028 antibodies co-immunoprecipitated BamA and BB0324 (Figure 4). However, none of the three proteins were detected in the Thio co-immunoprecipitation experiment control sample (Figure 4, left lane of each panel). Additionally, when immunoprecipitated proteins from all experiments were probed with antibodies to Lp6.6, which is a lipoprotein known to be localized to the inner leaflet of the borrelial OM [54], there was no detectable co-immunoprecipitation of Lp6.6 (Figure 4, bottom panel). The Lp6.6 immunoblot data demonstrated that an unrelated lipoprotein was not immunoprecipitated, which helped to confirm the specificity of the immunoprecipitation assays. The combined data indicate that BamA physically associates with BB0324 and BB0028.

### BamA expression is required for interaction with BB0324 and BB0028

**Figure 5:**
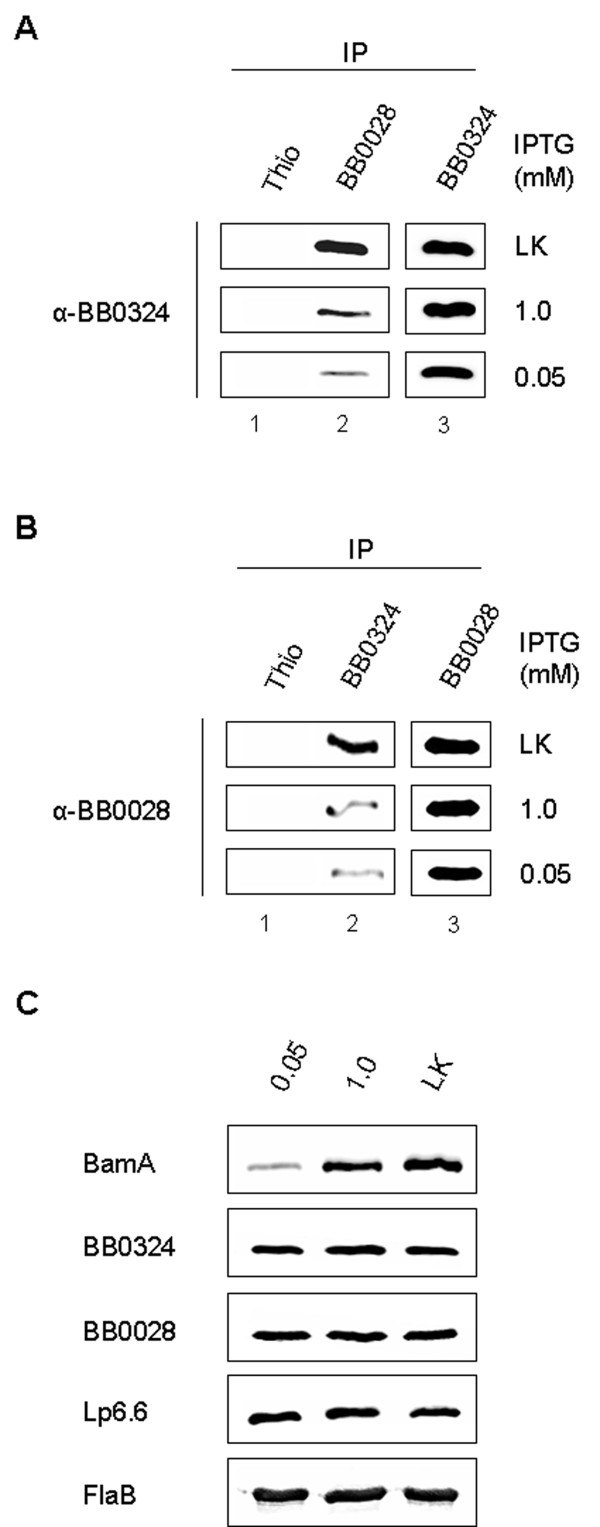
**BamA is required for efficient BB0324-BB0028 interactions**. Protein lysate from *B. burgdorferi *strain *flacp*-795-LK cultures (grown in 0.05 and 1.0 mM IPTG) and the parental strain B31-A3-LK cultures (grown in IPTG-deplete media) was used for co-IP using anti-Thio, anti-BB0324, and anti-BB0028 polyclonal antibodies (indicated above panels). Equal amounts of each co-IP elution were subjected to SDS-PAGE and immunoblot analysis. **A**. Anti-BB0324 immunoblots of the various co-IP elutions from the parental B31-A3-LK cultures (LK; top panel), *flacp*-795-LK cultures cultivated in 1.0 mM IPTG (middle panel), and *flacp*-795-LK cultures cultivated in 0.05 mM IPTG (bottom panel). **B**. Co-IP elutions were immunoblotted as in A, except with anti-BB0028 antisera. **C**. BamA depletion does not affect total cellular levels of BB0324 or BB0028. Prior to the cell lysis and solubilization procedure, spirochetes from each culture condition were washed and prepared as whole-cell lysates (WCL). Equal amounts of WCL (generated from 4 × 10^7 ^organisms) were subjected to anti-BamA immunoblot analysis in order to confirm the *flacp*-795-LK regulatable phenotype. The WCL were also immunoblotted with BB0324, BB0028, and Lp6.6 antisera to determine if cellular levels of each protein were affected by BamA depletion. A FlaB immunoblot is included to ensure equal loading of the *B. burgdorferi *WCL samples.

Although the above co-immunoprecipitation data indicated that BB0324 and BB0028 specifically interact with BamA, it was still unclear if BB0324 and BB0028 interacted with each other. We therefore wanted to determine if native BB0324 and BB0028 form their own complexes in *B. burgdorferi*, or if they interact only in the presence of BamA as constituents of the larger BAM complex. To examine this issue, we utilized the regulatable *B. burgdorferi *strain (*flacp*-795-LK) that was engineered to express an IPTG-inducible chromosomal *bamA *gene. We previously illustrated that in low concentrations of IPTG (0.05 mM), total cellular levels of BamA protein were dramatically reduced, and as a result, *B. burgdorferi *OM preparations contained reduced levels of OMPs [32]. By performing immunoprecipitation experiments with *flacp*-795-LK cultivated in a low concentration (0.05 mM) or high concentration (1.0 mM) of IPTG, we were able to observe the effects of BamA depletion on the BamA-BB0324-BB0028 interactions. As shown by immunoblot analysis, BamA depletion resulted in less BB0324 being immunoprecipitated by BB0028 antibodies as compared to the parental B31-LK strain (Figure 5A, lane 2, compare middle and bottom panels to top panel). Similarly, BamA depletion also resulted in less BB0028 being immunoprecipitated by BB0324 antibodies as compared to the parental B31-LK strain (Figure 5B, lane 2, middle and bottom panels versus top panel). However, it should be noted that there was no detectable difference in the levels of BB0324 or BB0028 expression after BamA depletion (see lane 3, Figure 5A and 5B). These data indicate that the loss of BamA did not affect the amount of BB0324 or BB0028 protein being expressed in the *flacp*-795-LK or parental LK strains. Consistent with this observation, whole-cell lysates (WCL) from the *flacp*-795-LK strain grown in 0.05 mM or 1.0 mM IPTG and the parental LK strain contained similar levels of BB0324 and BB0028 as shown in Figure 5C. The combined data revealed that BamA depletion does not affect expression of BB0324 or BB0028, but instead causes a decrease in the amount of BB0324 that is immunoprecipitated with BB0028, and also causes a decrease in the amount of BB0028 that is immunoprecipitated by BB0324. Thus, the BB0324 and BB0028 interactions with BamA appear to be severely affected by the loss of BamA expression, which also indicates that they require BamA in order to efficiently form the larger BAM complex.

### BB0324 and BB0028 are outer membrane-associated subsurface proteins

**Figure 6:**
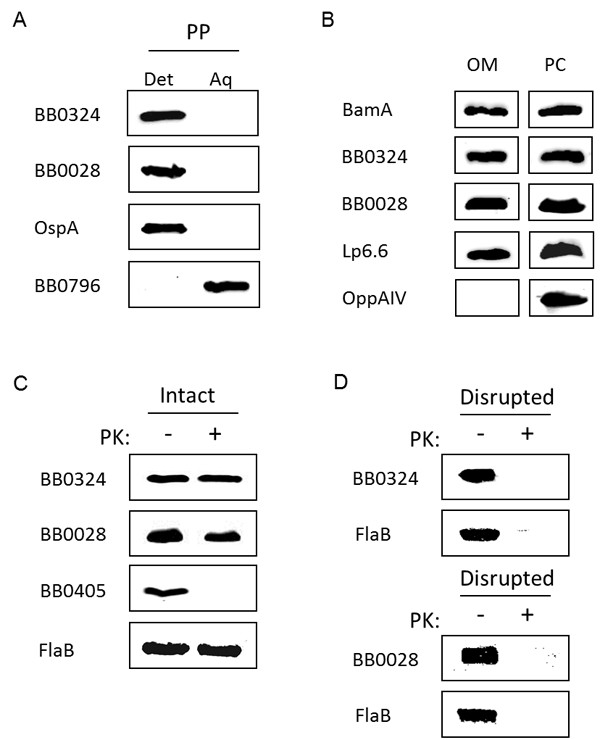
**Cellular localization of BB0324 and BB0028**. **A**. BB0324 and BB0028 are integral membrane proteins. Whole-cell lysates of *B. burgdorferi *B31 were subjected to Triton X-114 phase partitioning, and equal amounts of the detergent-enriched and aqueous phases were subjected to SDS-PAGE and immunoblot analysis with rat anti-BB0324 and rabbit anti-BB0028 antisera. To ensure proper phase separation, a known detergent phase protein and a soluble aqueous phase protein, OspA and BB0796, respectively, were included as controls. **B**. BB0324 and BB0028 are localized to the *B. burgdorferi *OM. OM and PC fractions from *B. burgdorferi *B31-A3-LK cells were isolated as described in Methods. Whole-cell equivalents from each fraction were subjected to SDS-PAGE and immunoblot analysis using BB0324 or BB0028 antisera. For positive controls, fractions were immunoblotted with antibodies against BamA and the known OM lipoprotein Lp6.6, which is anchored to the inner leaflet of the *B. burgdorferi *OM. To verify OM purity, fractions were also immunoblotted with antibodies against the inner membrane lipoprotein OppAIV. **C**. BB0324 and BB0028 are subsurface proteins. Whole-cell lysates of *B. burgdorferi *B31 cells were either mock-treated (-) or proteinase K-treated (+) before being immunoblotted with BB0324 or BB0028 antisera. As a positive control for PK activity, samples were probed with antibodies to BB0405, a known surface-exposed OMP. The mock-treated and the PK-treated samples were also immunoblotted with rabbit anti-FlaB antibodies to ensure equal loading. **D**. Subsurface BB0324 and BB0028 proteins are degraded by proteinase K. *B. burgdorferi *cell membranes were disrupted with detergent and lysozyme prior to incubating the lysates in the absence (-) or presence (+) of proteinase K. Samples were immunoblotted using antibodies to BB0324, BB0028, or FlaB (a known periplasmic protein).

Currently, all known accessory proteins of *E. coli *BAM complex, besides BamA, are lipoproteins anchored to the inner leaflet of the OM [7,10,18]. Therefore, we next examined whether both BB0324 and BB0028 are localized to the periplasmic leaflet of the OM. To begin our cellular localization assays, we first performed Triton X-114 (TX-114) phase partitioning studies with *B. burgdorferi *cells to determine if BB0324 and BB0028 are amphiphilic. As shown in Figure 6A, both BB0324 and BB0028 partitioned exclusively into the detergent-enriched fraction, which is characteristic of amphiphilic proteins. Additionally, a known membrane-anchored lipoprotein (OspA) and a soluble protein (BB0796) were used as detergent phase and aqueous phase controls, respectively.

We next examined the cellular location of BB0324 and BB0028 to confirm their presence in the OM. As shown in Figure 6B, BB0324 and BB0028 were detected in the isolated OMs of *B. burgdorferi*, demonstrating that both proteins are localized to the OM. Cell fractions were also probed with antibodies to the OM-localized BamA and Lp6.6 proteins, as well as to the IM-anchored OppAIV lipoprotein, to verify OM specificity and purity.

To determine if BB0324 or BB0028 are anchored to the periplasmic leaflet of the OM, we next incubated whole *B. burgdorferi *cells in the presence or absence of proteinase K (PK). These experiments revealed that there was no difference between mock- or PK-treated samples when probed with anti-BB0324 or anti-BB0028, indicating neither protein is surface-exposed (Figure 6C). As controls for PK activity and OM integrity, lysates from the mock- and PK-treated cells were also probed with antibodies against the surface-localized BB0405 protein [32,39] and the periplasmic FlaB protein, respectively. Importantly, when cell membranes were disrupted prior to PK treatment, BB0324 and BB0028 were both degraded in the presence of PK (Figure 6D) confirming that these proteins are subsurface proteins and not intrinsically resistant to PK activity.

## Discussion

### Current working model for the *B. burgdorferi *BAM complex

The bacterial beta-barrel assembly machine, or BAM, is a multiprotein OM complex that is composed of the essential integral OMP BamA, as well as a number of conserved and nonconserved accessory lipoproteins that are anchored to the inner leaflet of the OM [15,18,19,30,31]. To date, few BAM complexes have been studied, and since only those from proteobacteria have been characterized, it is yet to be determined what elements of various BAM complexes are conserved between different bacterial groups. In this study we report that the diderm spirochete, *B. burgdorferi*, also contains an OM-localized BAM complex, which is composed of BamA and at least two accessory lipoproteins, BB0324 and BB0028. Additionally, co-immunoprecipitation experiments using a BamA regulatable *B. burgdorferi *mutant strain indicated that BamA is required for efficient association of BB0324 and BB0028. Further cellular localization assays indicated that both BB0324 and BB0028 are OM anchored subsurface lipoproteins, although only BB0324 is predicted to be an ortholog to a currently identified BAM accessory lipoprotein (*i.e*., the *N. meningitidis *BamD lipoprotein).

As determined from our initial immunoprecipitation experiments with *B. burgdorferi *strain B31-MI, the BB0324 and BB0028 proteins associate specifically with BamA as a heterooligomeric OM protein complex (see Figure 4). Additional data from the BamA regulatable mutant provided further insight into the BamA-BB0324-BB0028 interactions. When the *bamA *IPTG-regulatable strain was cultivated in decreasing concentrations of IPTG (1.0 or 0.05 mM IPTG) it was immediately apparent that the BamA and BB0324/BB0028 associations were dramatically affected as compared to the parental, wildtype strain B31-LK (see Figure 5A and 5B). Although these data are insufficient to provide conclusions on the detailed organization of the BAM complex, it is apparent that BB0324 and BB0028 do not efficiently co-immunoprecipitate each other when BamA is depleted. These data suggest that BB0324 and BB0028 do not readily associate in *B. burgdorferi *without the presence of BamA, and that they likely come together only to form the functional BAM complex. However, the molecular architecture of the *B. burgdorferi *BAM complex is still unknown, and it is unclear what specific interactions create the BamA-BB0324-BB0028 complex. In our model, BB0324 and BB0028 may associate indirectly through individual direct contacts with BamA. Alternatively, BB0324 and BB0028 may bind directly with each other, where only one of them binds BamA. Further experiments using *B. burgdorferi bb0324 *and *bb0028 *partial and/or full deletion mutants (or IPTG regulatable mutants if they are found to be essential) should help to clarify the molecular architecture and binding partners within the BAM complex.

### Potential interactions between the periplasmic BamA polypeptide transport-associated (POTRA) domains and the BB0028 and BB0324 accessory lipoproteins

The periplasmic N-terminus of BamA contains five repeating POTRA (polypeptide transport-associated) domains, which have been designated P1-P5 [55], that are responsible for the BamA-accessory lipoprotein interactions [10,56]. It has been shown in *E. coli *that deleting any of the POTRA domains other than P1 results in disruption of accessory lipoprotein interactions [57]. Similar to the *E. coli *BAM accessory lipoproteins, it is likely that BB0324 and BB0028 also associate with BamA through POTRA domain contacts. Future co-immunoprecipitation experiments with different *B. burgdorferi *BamA POTRA domain mutants as well as BB0324, and/or BB0028 mutants will help clarify exactly which POTRA domains are needed for BB0324 and BB0028 accessory protein binding.

### BB0324 is a putative BamD ortholog with a truncated C-terminus

BlastP searches and sequence analyses indicate that the BB0324 protein is a putative *B. burgdorferi *BamD ortholog. BamD is predicted to be ubiquitous in diderm bacteria [10,15,21], and it appears to be both essential for cell survival and central to the function of the BAM complex, as demonstrated in *E. coli *and in *N. meningitidis *[18,21,25,30,58]. It is predicted that all BamD orthologs possess N-terminal TPR domains [15], and in *E. coli *and *N. meningitidis*, BamD appears to contain two (see Figure 2). Although such structural features are still predicted for *E. coli *and *N. meningitidis*, a recently-determined crystal structure from the *Rhodothermus marinus *BamD confirms the presence of TPR domains within this protein [59]. Although TPRs form a characteristic helix-loop-helix structure, their propensity for sequence variation is likely a reason that we were initially unable to identify a BamD ortholog in *B. burgdorferi*, even though BB0324 contains consensus TPR sequences [27-29]. In addition, BB0324 is considerably smaller than the BamD proteins currently identified in other bacteria. The putative borrelial BamD lipoprotein has a predicted MW of ~14 kDa, which is less than half the size of proteobacterial BamD proteins from *E. coli, N. meningitidis*, and *C. crescentus*. Interestingly, it has been proposed that the TPR domain region fulfills the major functional requirements for BamD (*i.e*., binding OMPs and/or interacting with BAM components), and that the TPRs may be the only essential feature of the BamD proteins [10,30]. This idea has been discussed in previous reports, and it originates from the discovery of a viable transposon mutant of the *Neisseria gonorrhoeae *BamD protein, also known as ComL [58]. As noted by Volokhina *et al*., this truncated mutant contains only 96 amino acids of the mature 267-residue protein, indicating that the ComL N-terminus, which comprises the TPR motifs, is sufficient for viability [30,58]. Although viable, the ComL mutant displayed reduced colony size and was deficient in transformation competency [58]. Similarly, an *E. coli *transposon mutant lacking only the C-terminal 19 amino acids of the 245-residue BamD protein was also viable but had greatly reduced BamD function [18,21]. These phenotypic characteristics suggest that the BamD C-terminus, although nonessential, fulfills some functional requirement for *Neisseria *and for *E. coli *(and likely for other proteobacteria) that is either unnecessary for *B. burgdorferi*, or is provided by a different protein. Interestingly, it has been shown that the C-terminus of the *E. coli *BamD binds BamC and BamE, and is therefore important for the stability of this part of the BAM complex [11,19,21,24,59]. Thus, a truncated *B. burgdorferi *BamD may simply be the result of this organism having no requirement for an extended C-terminal region to interact with additional accessory lipoproteins such as BamC or BamE, since we were not able to identify other accessory lipoproteins in *B. burgdorferi*.

## Conclusions

In the current study, we have identified two accessory components of the *B. burgdorferi *BAM complex. Based on the knowledge gained from studying other proteobacterial organisms, it is possible that *B. burgdorferi *contains one or more other BAM accessory lipoprotein components in addition to BB0324 and BB0028 that are still unidentified. As indicated by BN-PAGE in Figure 1A, multiple high molecular weight (MW) complexes containing BamA are present between approximately 148 kDa and over 1,000 kDa. These data accommodate the possibility that additional protein species may be co-migrating with BamA, especially since the smallest of the two most prominent bands, which migrates at ~200 kDa, has an approximate MW that is larger than the expected MW of BamA, BB0028, and BB0324 combined (~144 kDa). Alternatively, these large protein complexes may contain multiple copies of the same protein, such as multiple BB0324 molecules, and/or be homo-oligomers of the entire BAM complex. It should be noted, however, that *B. burgdorferi *contains a relatively small number of integral OMPs (at least 10-fold fewer) compared to *E. coli *[60,61]; hence, it may require a less complicated BAM complex system for OMP assembly. Indeed, Silhavy and coworkers proposed that the major function of the nonessential *E. coli *BamB, BamC, and BamE lipoproteins is most likely to increase efficiency of OMP assembly, or to stabilize the complex, since individual mutants were viable and showed relatively mild assembly defects [11,19,26]. It is, therefore, possible that an OM with a more limited OMP repertoire, such as that of *B. burgdorferi*, does not necessitate additional BAM complex members to provide the essential functions for complete OM biogenesis. In this regard, it is tempting to speculate that the *B. burgdorferi *BAM constituents identified here constitute a "minimal" bacterial BAM complex, which can now be further studied as a model system to not only further our understanding of *B. burgdorferi *OM biogenesis, but also to contribute to our current knowledge of bacterial OM biogenesis in general.

## Authors' contributions

TL carried out the experiments for Figures 2, 3, 4, 5 and 6A-C and drafted the initial manuscript. MK participated in the design of the studies and performed experiments for 6D and provided intellectual input and editing assistance for the manuscript. XY and UP provided the data for Figure 1. DA conceived of the study, participated in its design and coordination, and helped to draft and edit the manuscript. All authors read and approved the final manuscript.
